# Supplementary material for: Predicting gene expression from histone marks using chromatin deep learning models depends on histone mark function, regulatory distance and cellular states
Source: Nucleic Acids Res. 2024 Dec 11;53(4):gkae1212. doi: 10.1093/nar/gkae1212 (PMC11879020; doi:10.1093/nar/gkae1212)
Supplement: gkae1212_Supplemental_File [file gkae1212_supplemental_file.docx]

| **Epigenome ID (EID)** | **Shorthand name** | **GROUP** | **Epigenome Mnemonic** | **ANATOMY** | **TYPE** |
| --- | --- | --- | --- | --- | --- |
| E118 | Hepatocellular carcinoma | ENCODE2012 | LIV.HEPG2.CNCR | LIVER | CellLine |
| E116 | Lymphoblastoid | ENCODE2012 | BLD.GM12878 | BLOOD | PrimaryCulture |
| E114 | Lung carcinoma | ENCODE2012 | LNG.A549.ETOH002.CNCR | LUNG | CellLine |
| E087 | Pancreatic islets | Other | PANC.ISLT | PANCREAS | PrimaryTissue |
| E066 | Liver | Other | LIV.ADLT | LIVER | PrimaryTissue |
| E016 | HUES64 ESC | ESC | ESC.HUES64 | ESC | PrimaryCulture |
| E007 | H1 derived NPC | ES-deriv | ESDR.H1.NEUR.PROG | ESC_DERIVED | ESCDerived |
| E006 | H1 derived MSC | ES-deriv | ESDR.H1.MSC | ESC_DERIVED | ESCDerived |
| E005 | H1 derived trophoblast | ES-deriv | ESDR.H1.BMP4.TROP | ESC_DERIVED | ESCDerived |
| E004 | H1 derived mesendoderm | ES-deriv | ESDR.H1.BMP4.MESO | ESC_DERIVED | ESCDerived |
| E003 | H1 ESC | ESC | ESC.H1 | ESC | PrimaryCulture |

**Supplementary Table 1 Roadmap information on cell types.** ID and metadata for cell types taken from Roadmap.

**Supplementary Fig. 1 The performance of the promoter model is improved by including a larger region around the transcriptional start site (TSS) rather than a more focused, functional region.** Promoter model performance (brown) with a receptive field of 6,000 base-pairs symmetrical around the TSS and a smaller receptive field model, with the same architecture, including known functional regions in the promoter (blue). Performance (y-axis) is the Pearson correlation coefficient on the blind test sets for H3K4me3, the promoter model’s top performing mark. The whiskers represent the standard deviation across the different cell types and the 4-fold cross-validation.

**Supplementary Fig. 2 Performance of promoter model by cell type and histone mark.** Violin plot of the model’s performance on histone marks measured by Pearson correlation coefficient on the blind test sets. Here, the cell type performance is coloured by the cell state - Embryonic stem cell (ESC), ESC derived cell, adult primary tissue or cancer cell line. Splitting the performance by cell type and histone mark removes the multimodal distribution noted in **Fig. 2b**.

**Supplementary Fig. 3 Histone mark activity and relation to performance of promoter model.** (a) Average histone mark activity for the 6,000 base-pairs around the transcriptional start site of the training genes across all cell types and folds (free y-axis). Relationship between promoter model’s performance and the mean histone mark activity in the training set (b) and the test set (c). Since there isn’t positive relationships for all marks, b-c highlight how the promoter model’s performance was not just a result of the quantity of histone mark signal observed, rather that it learnt histone mark and cell type-specific regulatory relationships.

**Supplementary Fig. 4 Performance of promoter model by transcript length.** Correlation between the model performance (y-axis) and the average transcript length (x-axis , in base-pairs) of all exons for each gene across all cell types and k-fold. This is split by histone marks. The average transcript length of each gene was taken from Ensembl.

**Supplementary Fig. 5 Distal model’s correlation of histone marks’ function and performance by cell state.** (a) Correlation matrix of the distal model’s performance by the different histone marks across each cell type and cross-validation k-fold. The distinct group of active marks from the promoter model, highlighted in blue, was also replicated for the distal model. Bars along the y-axis correspond to the hierarchical clustering dendrogram. (b) Violin plot of performance measured by Pearson correlation coefficient on the blind test sets grouped by cell state and histone mark levels. Significance based on Mann-Whitney U-Test with false discovery rate multiple test correction where p-value indicators: * < 0.05, ns >= 0.05. (c) Correlation between the histone mark levels (average log2-transformed, read depth in the full 40,000 base-pairs around the transcriptional start site) and model performance for each cell type, k-fold combination. Split by repressive and active marks and highly and lowly expressed genes.

**Supplementary Fig. 6 Performance of distal model in gene expression predictions from two histone mark pairs with H3K27me3.** Performance for combinations of all pairs with H3K27me3, a repressive mark. The range of values represent the standard deviation across the different cell types and the 4-fold cross-validation. Data is averaged at the level of cell type and k-fold. The red dashed line and shaded box shows the model’s mean performance and standard deviation when trained on all seven histone marks together. Significance based on false discovery rate (FDR) multiple test correction where p-value indicators: **** < 1e-4, *** < 1e-3 and ** < 1e-2.

**Supplementary Fig. 7 Performance improvement of distal model from two histone mark pairs with H3K27me3 in bivalent genes.** Performance improvements for combination distal model over the single mark distal model for the top three, activating histone marks with H3K36me3. The range of values represent the standard deviation across the different cell types and the 4-fold cross-validation. Data is averaged at the level of cell type and k-fold. Performance improvement is split by mark, cell type group and bivalent and non-bivalent genes. Bivalent genes are defined as those with both an active histone mark signal and repressive - H3K36me3 signal in the gene promoter region (both above the median for the cell type). Plot is also split by highly and lowly expressed genes defined in the same way as for the histone mark level, above or below the median for the cell type.

**Supplementary Fig. 8 Correlation across 4 fold cross-validation models in the *in silico* perturbation analysis.** (a) Correlation for Chromoformer, the histone mark model on matched genes and histone mark perturbation loci and level across the 4-fold, cross-validation models. All models showed high concordance (Pearson R=0.96). (b) Correlation for Borzoi, a DNA model on matched genes and *in silico* mutagenesis levels across the 4-fold, cross-validation models. The same cell types and receptive field were inspected for correlation to chromoformer to make for a fair comparison. All models also showed high concordance (Pearson R=0.94) but slightly less than the histone mark model approach.

**Supplementary Fig. 9 Effect of *in silico* histone mark perturbation on expression by cell state.** The effect on quantile predicted expression (y-axis) of changing the proportional levels of measured histone mark activity (x-axis) for all cell types and genes, averaged across the four k-fold models. The distal model trained on a single histone mark was used to measure the effects of a perturbed active mark - H3K27ac or a repressive mark - H3K27me3. The effect of distance on expression change is shown when the histone mark activity is completely removed at a specific locus. The distribution of all gene expression changes in all folds is split into 20 quantile bins where each line represents a cell type, grouped into cell states. H1 derived MSC is highlighted for its unique response in the repressive mark, ESC derived group.

**Supplementary Fig. 10 Effect of *in silico* histone mark perturbation on expression by histone mark.** The effect on predicted expression (y-axis) of changing the proportional levels of measured histone mark activity (x-axis) for all cell types and genes, averaged across the four k-fold models. Two different active histone marks were tested, a mark with known distal regulatory effects - H3K27ac, and a mark with local regulatory effects to the TSS - H3K4me3. The effect of distance on expression change is shown when the histone mark activity is completely removed at a specific locus. H3K4me3 has higher predicted change in expression in the TSS where H3K27ac has higher downstream and in the gene body.

**Supplementary Fig. 11 Improvement of model over alternative loci prioritisation methods for fine-mapped eQTL enrichment.** Upstream and downstream *in silico* histone mark perturbation experiments from the active model (trained on H3K27ac) were sorted into deciles based on their predicted change in expression and the top decile used; ‘Model’, to compare against (a) the cell type-specific region of max histone mark activity; ‘H3K27ac max activity’, (b) cell type-specific Hi-C data upstream of matching genes; ‘Hi-C’ and (c) upstream and downstream up to 6,000 base-pairs away ‘<=6Kbp’. All were tested for enrichment of fine-mapped eQTL interactions in matched cell types (y-axis) and compared against bootstrap sampling random upstream loci 10,000 times to generate p-values of enrichment (x-axis). Lower p-values for the model indicate greater enrichment found in more tests against randomly selected loci than the alternative method. For Hi-C Lung (b), <=6Kbp Cerebellum & Hippocampus and Lymphocytes (c), both the model and alternative method obtained equivalent p-values. Note, no fine-mapped eQTL SNPs were located <=6Kbp in the Pancreas so no dot is shown for the alternative model in (c).

**Supplementary Fig. 12 Embedding self-attention weights for the *in silico* histone mark perturbation of *DSTN* in a lung carcinoma cell line.** Upstream *in silico* histone mark perturbation of *DSTN* in the lung carcinoma cell line using the distal model trained on the H3K27ac signal. The experimental H3K27ac signal is shown along with the embedding self-attention weights for the distal model, before and after ablation of the H3K27ac signal in the highlighted region. The fine-mapped SNP rs611572 from the UK Biobank population eQTL analysis in a matched cell type is also shown. This region was chosen as the *in silico* perturbation analysis identified it in the top decile of predicted effects on expression for this cell type.
